# A Rare Case of Hereditary Hemochromatosis Presenting As Hyperbilirubinemia

**DOI:** 10.7759/cureus.65098

**Published:** 2024-07-22

**Authors:** Anish Chitnis, Kunal Modi, Jagannath S Dhadwad, Mallika Agarwal, Chandan Dash

**Affiliations:** 1 General Medicine, Dr. D. Y. Patil Medical College, Hospital, and Research Centre, Pune, IND; 2 Pathology, Dr. D. Y. Patil Medical College, Hospital, and Research Centre, Pune, IND

**Keywords:** hfe, phlebotomy, iron deposits, hyperbilirubinemia, hemochromatosis

## Abstract

Hemochromatosis is a condition marked by excessive iron accumulation, causing dysfunction in various organs. A 50-year-old woman, previously in good health, reported abdominal pain and yellowing of the skin and eyes for one month. Upon examination, she exhibited widespread jaundice, leg swelling, and abdominal distention. Her total bilirubin level was 24.52 mg/dL at admission, indicating hyperbilirubinemia. Imaging studies, including USG and CT scans, revealed mild to moderate ascites and altered liver texture. Elevated serum ferritin (1443 ng/mL) and transferrin saturation (84%) suggested iron overload. A liver biopsy confirmed the presence of iron deposits in hepatocytes, leading to a diagnosis of hemochromatosis. Genetic testing was negative for the C282Y and H63D mutations, resulting in a diagnosis of non-homeostatic iron regulator (non-HFE) related hereditary hemochromatosis. The patient began weekly phlebotomy and was monitored regularly, with a liver transplant being considered as a potential treatment.

## Introduction

Hemochromatosis is a disorder characterized by the accumulation of iron, leading to multi-organ dysfunctions. Iron absorption is typically strictly controlled since the body cannot eliminate excess iron [1]. Previously considered a single disease, hemochromatosis is now recognized as a genetically diverse iron-storage disorder. Despite this diversity, the condition follows a common metabolic pathway, resulting in abnormal intracellular release of iron. This leads to increased intestinal iron absorption and excessive iron deposition in parenchymal cells, causing tissue damage and organ failure [2].

Hereditary hemochromatosis is primarily caused by mutations in the homeostatic iron regulator (HFE) gene, which is closely linked to the HLA-A locus on chromosome 6p. Individuals homozygous for this mutation are at a higher risk for iron overload, accounting for 80-90% of hereditary hemochromatosis cases [2]. These mutations lead to increased iron absorption despite normal dietary intake. The most common HFE gene mutations are C282Y and H63D [3].

Secondary iron overload can result from conditions such as thalassemia or sideroblastic anemia, where erythropoiesis is increased but ineffective. This leads to substantial iron deposits in tissues similar to those seen in hemochromatosis. Approximately 40,000 beta-thalassemia patients are born annually in Southeast Asian countries [2].

## Case presentation

A 50-year-old previously healthy female presented at the medicine OPD with complaints of yellowish discoloration of the sclera and skin for the last 30 days, which progressively worsened over one month. In the last two weeks, she also complained of dull, aching pain in the right upper quadrant of her abdomen, associated with vomiting, approximately two to three episodes per day. The vomit was non-projectile, bilious, and aggravated by food consumption. Additionally, she experienced generalized weakness, malaise, and lethargy over the last two weeks, without any fever. She noticed swelling over both lower limbs 15 days ago, which increased over the past seven days, accompanied by abdominal distension.

She had no complaints of chest pain, breathlessness, or palpitations. There were no bleeding manifestations such as melena, hematochezia, or hematemesis.

She was diagnosed with hypothyroidism two months prior and started on L-Thyroxine 25 mcg once daily. She was not taking any other medications, including herbal supplements. She had no history of hypertension, diabetes mellitus, ischemic heart disease, or tuberculosis and denied any alcohol consumption or smoking.

The patient was vitally stable with a pulse rate of 90 beats per minute, blood pressure of 110/70 mmHg, oxygen saturation of 97% on room air, and a respiratory rate of 18 cycles per minute. There was diffuse icterus noticeable over the skin of the face, palms, soles, and sclera with an orange-yellow tinge. Pitting edema was present over the shin of the tibia. There was no pallor, cyanosis, or clubbing.

On per abdominal examination, mild distension was seen with an inverted umbilicus, with no scars or sinuses visible. Fullness of the flanks was seen and shifting dullness was appreciated. There was no abdominal tenderness or organomegaly, and bowel sounds were audible.

On Day 1, tests for HIV, Hepatitis B surface antigen (HBsAg), and Hepatitis C virus (HCV) returned non-reactive. Laboratory tests on Day 1 showed normal hemoglobin levels with a normal total leukocyte count (TLC) and thrombocytopenia. Bilirubin levels were significantly elevated. Electrolytes, renal parameters, and serum lactate dehydrogenase (LDH) were within normal limits. The international normalized ratio (INR) was elevated and trending upward, requiring intervention (Table 1). Her Child Pugh score was 12 points, classifying her as Child Pugh class C, and her model of end stage liver disease (MELD) score was 35 points.

**Table 1:** Laboratory parameters. TLC: Total Leucocyte Count; AST: Aspartate Transaminase; ALT: Alanine Transaminase; ALP: Alkaline Phosphatase; Na: Sodium; K: Potassium; PT/INR: Prothrombin Time/International Normalized Ratio; gm/dl: Grams per deciliter; mg/dl: Milligrams per deciliter; mmol/L: Millimoles per liter; U/L: Units per liter; microL: Microliter.

Parameters	Day 1	Day 3	Day 7	Reference range
Hemoglobin	11.1 gm/dl	10.5 gm/dl	9.7 gm/dl	11.0-15.0 gm/dl
TLC	8600/microL	10900/microL	9300/microL	4000-10000/microL
Platelets	89000/microL	92000/microL	127000/microL	150000-410000/microL
Total bilirubin	24.52 mg/dl	24.27 mg/dl	23.47 mg/dl	0.22-1.20 mg/dl
Direct bilirubin	17.53 mg/dl	17.25 mg/dl	17.73 mg/dl	Upto 0.5 mg/dl
Indirect bilirubin	6.99 mg/dl	7.02 mg/dl	5.47 mg/dl	0.1-1.0 mg/dl
AST	121 U/L	135 U/L	146 U/L	8-43 U/L
ALT	136 U/L	96 U/L	83 U/L	7-45 U/ l
ALP	164 U/L	141 U/L	132 U/L	35-104 U/L
Na	142 mmol/L	135 mmol/L	136 mmol/L	136-145 mmol/L
K	4.38 mmol/L	4.29 mmol/L	4 mmol/L	3.50-5.10 mmol/l
Urea	40	27	20	17-49 mg/dl
Creatinine	1.2 mg/dl	1.10 mg/dl	1.13 mg/dl	0.6-1.2 mg/dl
Albumin	2.8 gm/dl	2.5 gm/dl	2.4 g/dl	3.5-5.2 g/dl
PT/INR	34.7/3.68	46.40/4.14	24.7/2.28	10.2-12.71 sec/ 0.85-1.15

Based on the clinical presentation and initial labs, a working diagnosis of acute decompensated liver disease secondary to infection, autoimmune conditions, or obstructive causes was made. These etiologies were initially considered given the short duration of symptoms and the patient's female gender. Accordingly, the following treatment plan was formulated:

The patient was started on IV antibiotics (Inj. Ceftriaxone 1 gram BD) empirically, diuretics (Tab. Furosemide 20 mg + Tab Spironolactone 50 mg BD) along with albumin supplementation to reduce the edema, Tab Ursodeoxycholic acid (300 mg TDS), and Tab Rifaximin (500 mg BD). Vitamin K supplementation was also given.

On Day 2, tests for Leptospira IgM, Dengue NS1 antigen/IgM and IgG, Weil Felix, Hepatitis A IgM, and Hepatitis E IgM were sent, which came back negative on Day 3.

A 2D ECHO showed a normal left ventricle with an ejection fraction of 60%, no regional wall motion abnormalities, and normal diastolic function.

Ultrasonography of the abdomen and pelvis performed on Day 2 showed altered echotexture of the liver with surface nodularity and a liver size of 12.7 cm. It also showed mild to moderate ascites.

Ascitic fluid routine microscopy performed on Day 2 showed a yellowish color, TLC- 110 per mm³, Albumin- 0.5 g/dL, LDH- 65 U/L, and protein- 0.7 gm%. Her SAAG (serum ascites-albumin gradient) was more than 1.1 with a low ascitic protein, which indicates cirrhosis.

**Figure 1:**
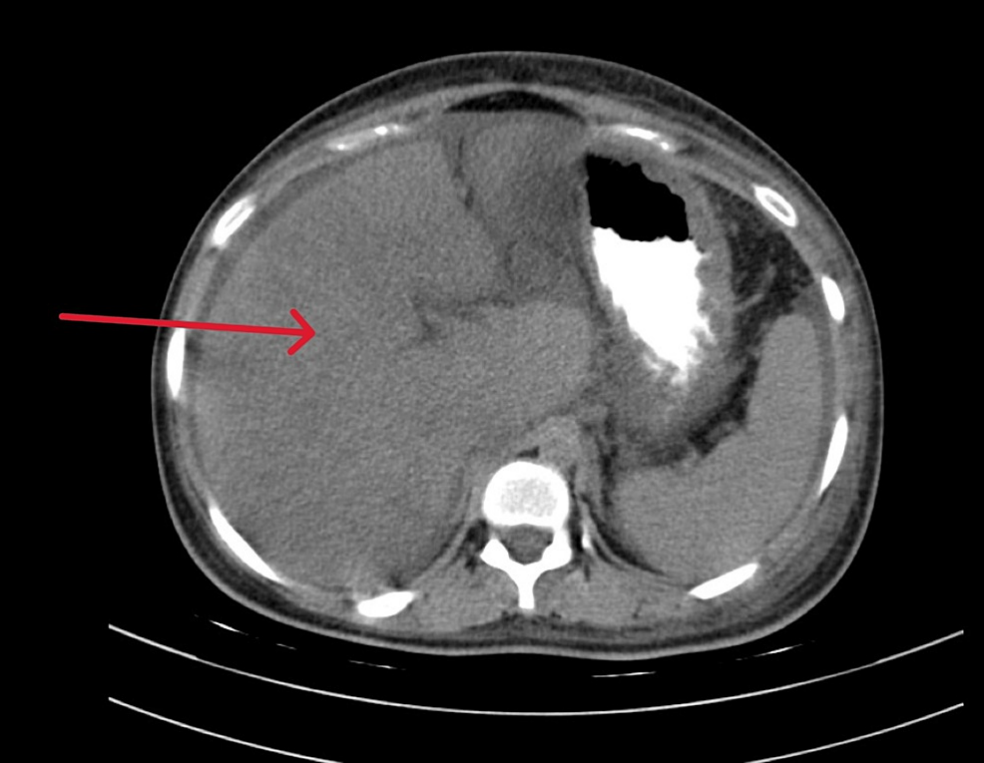
CECT of the abdomen and pelvis showing altered echotexture of the liver (indicated by the red arrow). CECT: Contrast-enhanced computed tomography.

**Figure 2:**
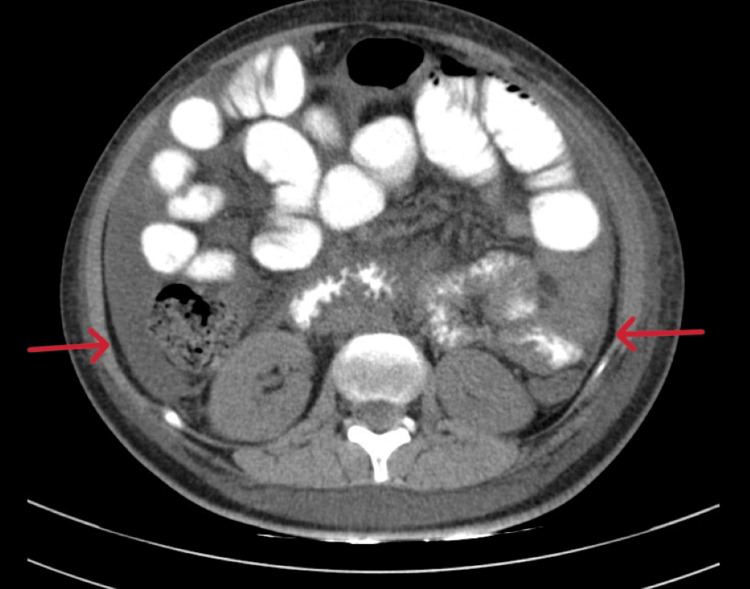
CECT of the abdomen and pelvis showing mild ascites (indicated by the red arrows). CECT: Contrast-enhanced computed tomography.

A CT scan (plain and contrast-enhanced) of the abdomen was done on Day 3, which showed altered echotexture of the liver and mild ascites (Figures 1-2).

Esophago-Gastro-Duodenoscopy showed no varices but mild portal-hypertensive gastropathy and antral gastritis were seen. A rapid urease test was positive for H. pylori.

Tab Amoxicillin (750 mg) + Tinidazole (500 mg) + Omeprazole (20 mg) BD was started in view of the positive Rapid urease test for H. pylori; and fresh frozen plasma was given in view of the markedly elevated INR.

On Day 4, direct Coombs test, indirect Coombs test, anti-nuclear antibody assay (ANA) by immunofluorescence, ANA blot, anti-smooth muscle antibody, and liver kidney microsomal antibody tests were sent, which all came back negative on Day 6.

Serum ceruloplasmin and 24-hour urinary copper were also sent and were within normal limits, along with iron studies which showed serum iron levels of 148 micrograms/dL (↑) (reference range 35-145 micrograms/dL), total iron-binding capacity (TIBC) - 176 micrograms/dL (↑) (reference range 250-450 micrograms/dL), transferrin saturation (TSAT) - 84% (↑↑) (reference range 20-50%) and serum ferritin - 1443 ng/mL (↑↑) (reference range 4.6-204 ng/mL) (Table 2).

**Table 2:** Iron studies. TIBC: Total iron binding capacity; TSAT: Transferrin saturation; ng/mL: Nanograms/milliliter.

Parameters	Values	Reference range
Serum iron	148 microgram/dl	35-145 microgram/dl
TIBC	176 microgram/dl	250-450 microgram/dl
TSAT	84%	20-50%
Serum ferritin	1443 ng/mL	4.6-204 ng/ml

**Figure 3:**
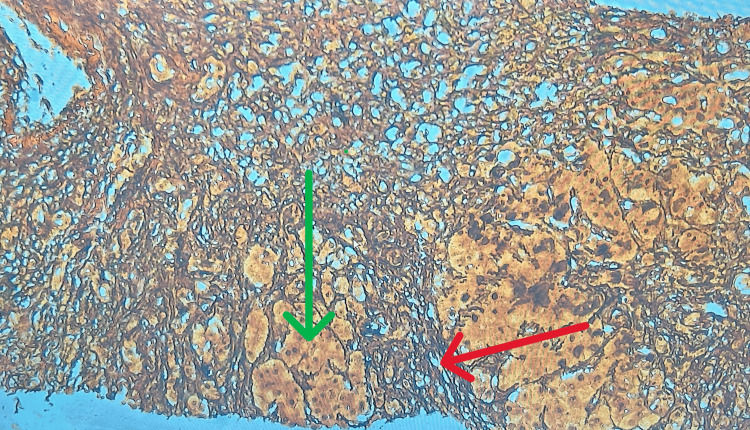
Liver biopsy stained with reticulin stain showing fibrosis (indicated by the red arrow) and surrounding hepatocytes (indicated by the green arrow) at 40x magnification.

**Figure 4:**
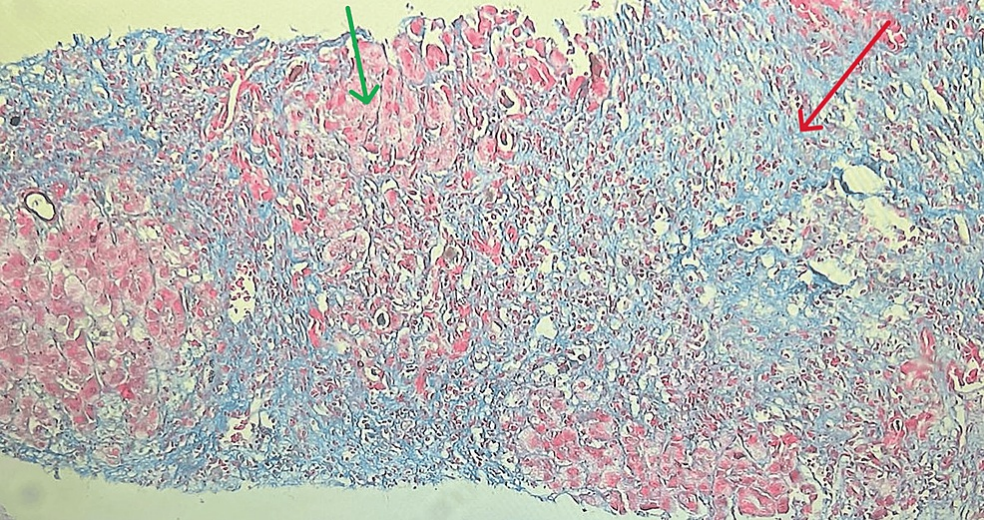
Liver biopsy stained with Masson trichrome showing fibrosis (indicated by the red arrow) with surrounding normal hepatocytes (indicated by the green arrow).

**Figure 5:**
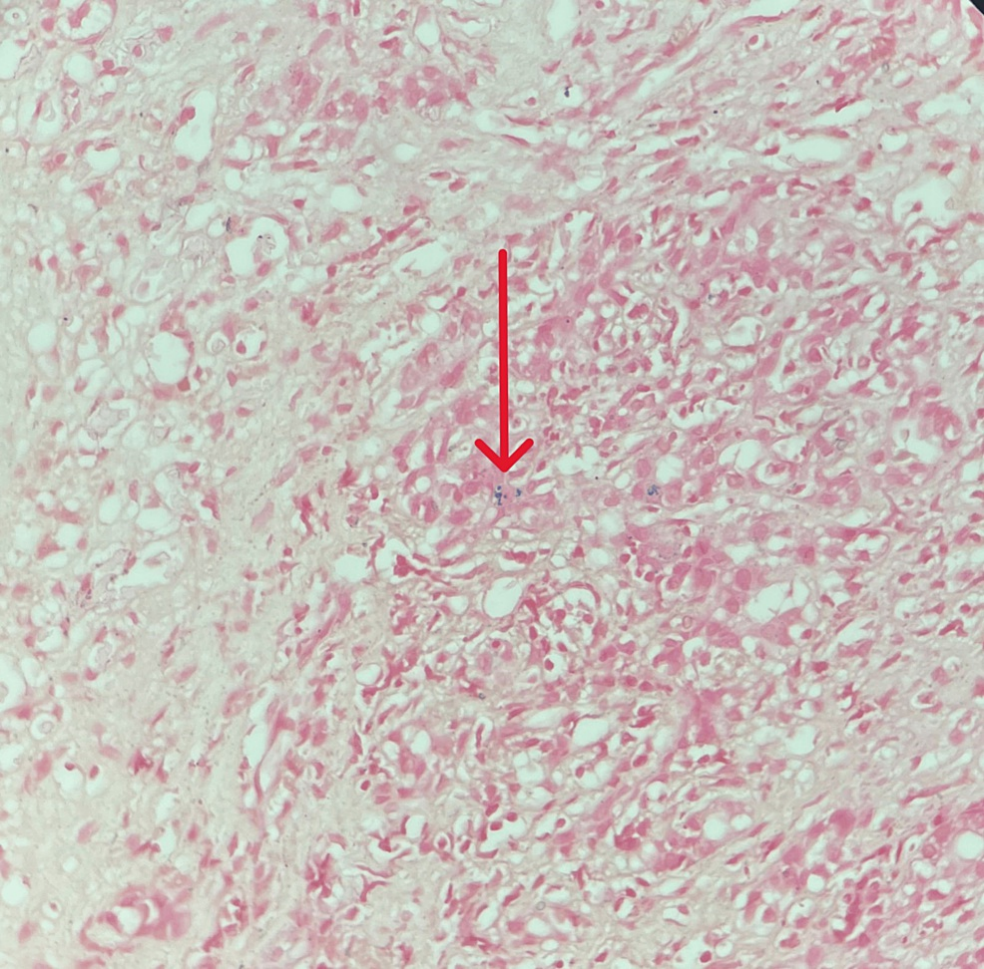
Liver biopsy stained with iron stain showing iron deposits in hepatocytes (indicated by the red arrow).

On Day 7, a liver biopsy was performed. Histopathological examination revealed moderate to severe interface hepatitis (grade 3, Batts-Ludwig system, suggestive of severe fibrosis) with stage 4 cirrhosis and focal hepatocytes containing iron (Figures 3-5).

Following this report, a genetic evaluation to check for common gene mutations associated with hemochromatosis, i.e., C282Y and H63D, was done which came out to be negative.

The patient was diagnosed with hemochromatosis, likely secondary to non-HFE mutations. The patient was started on phlebotomy once a week, and further evaluation was advised. A liver transplant is being considered.

## Discussion

Hereditary hemochromatosis is a common genetic disorder among white populations with an autosomal recessive inheritance. It has a prevalence of 1 in 300 to 500 individuals [4]. There are four types of hereditary hemochromatosis. Type 1, the classic form, is HFE-related and autosomal recessive, with a global prevalence [5]. Type 2 is divided into subtypes 2a (hemojuvelin gene mutations) and 2b (hepcidin gene mutations), also autosomal recessive, and usually manifests between 15 and 20 years of age [1]. Type 3 involves mutations in the transferrin receptor-2 gene and typically appears between 30 and 40 years [6]. Type 4, linked to ferroportin gene mutations, is an autosomal dominant disorder with an onset range from 10 to 80 years [1].

Men are affected by hemochromatosis 2-3 times more frequently than women, with symptoms typically emerging in the fifth decade of life for men and the sixth for women, due to menstruation-related iron loss [1].

Hereditary hemochromatosis results from homozygosity for an HFE protein mutation, leading to increased iron absorption. The HFE protein is crucial for regulating hepcidin production, the hormone responsible for iron regulation [7]. Normally, the body balances iron absorption and loss, maintaining 3-4 grams of iron content. In hemochromatosis, mucosal absorption exceeds the body's needs, reaching levels of 4 mg per day or more [2].

Hepcidin, produced by the liver, inhibits iron export from intestinal cells and macrophages. Its production is regulated by signals involving HFE, TFR2, and hemojuvelin. Mutations in HFE decrease hepcidin production, increasing dietary iron absorption [2].

Secondary hemochromatosis can occur due to conditions like erythropoietic hemochromatosis, which results from the overproduction of RBCs and their subsequent destruction, leading to iron deposits in tissues [1]. Hereditary aceruloplasminemia, a disorder caused by ceruloplasmin deficiency, also leads to iron overload in hepatocytes and other cell types [2].

Hemochromatosis affects multiple organs, including the thyroid, pancreas, liver, heart, gonads, skin, and pituitary glands. Approximately 70% of patients develop cirrhosis, significantly increasing the risk of hepatocellular carcinoma. Pancreatic iron deposition primarily manifests as diabetes in about 50% of symptomatic individuals. Cardiac symptoms arise from iron deposition in myocardial fibers, leading to congestive heart failure and arrhythmias, sometimes reversible by reducing iron stores. Skin hyperpigmentation occurs due to iron and melanin deposition [1].

A case reported by Anderson MB et al. involved a 65-year-old female with sickle cell disease and end-stage renal disease on regular dialysis. She presented with fatigue and breathlessness, with elevated serum bilirubin and liver enzymes. CT findings indicated cholelithiasis and hepatomegaly consistent with hemochromatosis, likely secondary to repeated blood transfusions [8].

Another case report by Yin X et al. involved a 34-year-old male with no history of diabetes or hypertension, presenting with right upper quadrant abdominal discomfort. Elevated serum ferritin and iron levels, along with MRI findings, indicated extensive liver and spleen iron deposition. Liver biopsy and genetic testing confirmed a mutation in the SLC40A1 gene, encoding the ferroportin-1 protein, indicating a rare autosomal dominant form of hereditary hemochromatosis [9].

Early initiation of phlebotomy might reverse the toxic effects of iron overload. Approximately 500 ml of blood can be removed weekly or even twice weekly depending on the initial iron stores, and this should continue until serum ferritin levels normalize. Following this, a maintenance therapy protocol can be established based on the patient's tendency for iron re-accumulation. Studies have shown a 66% survival rate in patients initiated on phlebotomy [10]. Hepatic fibrosis can improve following venesection, but hepatic cirrhosis is generally irreversible.

Patients with genetic hemochromatosis exhibit lower survival rates post-liver transplant compared to other recipients (53% versus 81% survival at 25 months) [11,12]. This decreased survival rate is primarily due to cardiac complications and sepsis, highlighting the importance of early diagnosis and iron removal therapy.

About one-third of patients undergoing liver transplantation for reasons other than genetic hemochromatosis present with hepatic iron deposition, and 10% have hepatic siderosis levels comparable to those in genetic hemochromatosis. HFE gene mutations are infrequent in this subgroup. Post-transplantation survival is significantly reduced in patients with hepatic iron overload [13].

## Conclusions

This is a case of hyperbilirubinemia presenting as a rare form of hemochromatosis. Hemochromatosis was suspected due to elevated serum ferritin and transferrin saturation levels, and the diagnosis was confirmed by liver biopsy. Genetic studies were negative for C282Y and H63D mutations, which are the common mutations seen in hemochromatosis. Further genetic studies were advised to investigate non-HFE types of mutations, but the patient's relatives declined any further investigations due to financial constraints. Secondary hemochromatosis was ruled out based on the history, as there were no blood transfusions or viral infections in the past. The patient was started on weekly phlebotomy and is under regular follow-up. This case represents a rare condition that presented as hyperbilirubinemia, highlighting the need for physicians to maintain a watchful eye.
